# Seasonal pattern of influenza activity in a subtropical city, China, 2010–2015

**DOI:** 10.1038/s41598-017-17806-z

**Published:** 2017-12-13

**Authors:** Xu-Xiang Liu, Yahong Li, Yibing Zhu, Juanjuan Zhang, Xiaoru Li, Junqing Zhang, Kefu Zhao, Mingxia Hu, Guoyou Qin, Xi-Ling Wang

**Affiliations:** 1 Hefei Center for Disease Control and Prevention, Anhui, China; 2 Department of Biostatistics, School of Public Health and Key Laboratory of Public Health Safety, Fudan University, 200231 Xuhui District, Shanghai, China; 3 Shanghai Key Laboratory of Meteorology and Health, Shanghai, China

## Abstract

Influenza seasonality study is critical for policy-makers to choose an optimal time for influenza vaccination campaign, especially for subtropical regions where influenza seasonality and periodicity are unclear. In this study, we explored the seasonality and periodicity of influenza in Hefei, China during 2010 to 2015 using five proxies originated from three data sources of clinical surveillance of influenza-like illness (ILI), laboratory surveillance of influenza and death registration of pneumonia and influenza. We combined both wavelets analysis and de-linear-trend regression with Fourier harmonic terms to estimate seasonal characteristics of epidemic phase, peak time, amplitude, ratio of dominant seasonality. We found both annual cycle of influenza epidemics peaking in December-February and semi-annual cycle peaking in December-February and June-July in subtropical city Hefei, China. Compared to proxies developed by ILI and death registration data separately, influenza proxies incorporated laboratory surveillance data performed better seasonality and periodicity, especially in semi-annual periodicity in Hefei. Proxy of ILI consultation rate showed more timeliness peak than other proxies, and could be useful in developing the early warning model for influenza epidemics. Our study suggests to integrate clinical and laboratory surveillance of influenza for future influenza seasonality studies in subtropical regions.

## Introduction

Influenza is a respiratory infection caused by influenza virus. Approximately 10–20% of the world’s population get infected with seasonal influenza virus annually, among which 3–5 million cases are severely ill and 250,000–500,000 cases die from influenza infections. As for China, annual influenza-associated excess mortality was 18.0 and 11.3 deaths per 100,000 population in northern and southern cities, respectively^1^. Although China is experiencing a substantial disease burden of influenza^2^, influenza vaccine coverage rate is only around 2.0% in 2016^3^. A large majority of the Chinese population are not protected by influenza vaccine^4^.

In response to the growing recognition that more needs to be done in preventing, monitoring and controlling influenza worldwide, influenza Surveillance has been identified to be extremely important by the World Health Organization (WHO) Global Agenda on Influenza Surveillance and Control^5^. China has established the influenza surveillance system in 2000, which is a centralized online system maintained by Chinese Center for Disease Control and Prevention (China CDC, Beijing), providing weekly reports from 193 sentinel hospitals located in 88 cities representing 30 provinces(except Tibet)^6,7^. The influenza surveillance network included both clinical and virologic surveillance for influenza. Data from influenza surveillance network has informed current understanding about the seasonality of influenza epidemics and characteristics of influenza cases. Various proxies or indexes have been developed using the surveillance data to represent influenza activity, while the performance of different proxies has never been compared^8,9^. Characteristics of epidemic peak time, amplitude and periodicity are key parameters to define seasonality. Most of studies estimate the parameters by setting a threshold for influenza epidemic and non-epidemic period^10,11^, while few were based on modeling techniques.

Research on influenza seasonality and periodicity is critical to guide control strategies for influenza epidemics. Lin^12^ conducted a logistic regression model together with a Fast Fourier transformation (FFT) to extract the periodic components of the time series. Serfling regression model were applied to assess the excess numbers of influenza-like illness cases attributed to influenza by Wang, etc^13^. There were still a lack of long-time surveillance data and relevant seasonality analysis, particularly in most subtropical cities of China.

Previous work has suggested intriguing differences in the seasonality and evolutionary dynamics of influenza between northern and southern China^14^. Three epidemiological regions characterized by distinct seasonality were identified in China. Northern provinces (latitudes >33°N) experiences winter epidemics^15^, southernmost provinces (latitudes <27°N) experiences peak activity in spring^12^, and provinces in the intermediate latitude experience an unclear but seemingly semi-annual periodicity with peak activities both in winter and summer. More detailed analysis of influenza pattern for regions in the intermediate latitude will provide evidence to choose the best influenza vaccination time for cities in the intermediate latitude. Our study aims to evaluate influenza seasonal pattern using five frequently-used proxies for influenza activity in the subtropical city of Hefei, Anhui province, 2010–2015.

## Method

### Influenza surveillance and death registry dataset

We obtained weekly reports of influenza-like illness (ILI) consultation rates and laboratory-confirmed influenza numbers, as well as total specimens tested, from Hefei CDC for the study period 2010–2015. Hefei has established the influenza surveillance system at the end of 2001, which is an online reporting system providing weekly reports from two sentinel hospitals: the First People’s Hospital and the Second People’s Hospital. In each sentinel hospital, nasopharyngeal swabs were collected from the first one or two ILI cases (defined as temperature ≥ 38 °C with either cough or sore throat, in the absence of an alternative diagnosis, according to WHO ILI standard case definition since October 2005) and placed in sterile viral transport medium for influenza virus testing, resulting in 10 to 15 specimens per hospital per week. Samples were stored at 4 °C to 8 °C and sent to Hefei CDC laboratory for test in 24 hours. The fluorescent real-time RT-PCR assay were performed to identify the types/subtype of influenza virus, following a standard protocol^14^. The fluorescent real-time RT-PCR assay, which was developed on the basis of conventional RT-PCR and has now been the main influenza virus screening method, is highly sensitive and accurate^16^. Firstly, we obtained cDNA from fluorescent RT-PCR, and then use fluorescent real-time RT-PCR for quantitative analysis. In addition, compared with the classical cell culture method for virus isolation with Madin-Darby canine kidney (MDCK) cells or specific pathogen free (SPF) chicken embryo, the procedure of fluorescent real-time RT-PCR is more simple and less time consuming. There are more detailed statement and comparison about the three methods for detection of influenza viruses in Zhang’s study^17^.

We chose the study period from 2010 as the quality of the surveillance data improved dramatically after the 2009 influenza pandemic. We obtained the weekly number of cases with death certificate coded as pneumonia and influenza (P&I, International Classification of Diseases, ICD10 J09-J18) and the whole population size of Hefei respectively from death registry and bureau of statistics (NBS) of Hefei.

### Definition for influenza proxies

Herein, three proxies incorporated laboratory surveillance data, one from clinical surveillance data, and another from death registration data were defined:

1. flu%: weekly proportion of specimens tested positive for influenza (flu% = number of specimens tested positive per week/ total number of specimens tested per week ${\rm{\times }}$ 100%). This proxy adjusted for weekly variation of specimens collected.
2. annflu%: proportion of specimens tested positive for influenza per week among total specimens tested positive per year (annflu% = number of specimens tested positive for influenza per week/ the sum of specimens tested positive for influenza per year ${\rm{\times }}$ 100%). This proxy adjusted for annual variation of specimens collected.
3. ILI rate: weekly ILI outpatient consultation rate (ILI rate = weekly number of ILI case /weekly total outpatient consultations ${\rm{\times }}$ 100%). Weekly ILI rate referred to the proportion of ILI cases among the total outpatient visits per week in two sentinel hospitals in Hefei.
4. ILI × flu%: product of ILI consultation rate and flu%. The proxy represented weekly rate of influenza cases among all ILI outpatient visits, by integrating the laboratory and clinical surveillance data.
5. P&I: weekly deaths rate of pneumonia and influenza (P&I = the number of deaths caused by pneumonia and influenza per week/population size ${\rm{\times }}$ 100% ${\rm{\times }}$ 10^4^). The value of P&I was magnified ten thousand time to be comparable with other four proxies.

### Wavelets analysis and seasonality regression

Wavelet analysis was commonly utilized to analyze time series that contain non-stationary power at many different frequencies. It is based on the wavelet transform which breaks the signal into a sum of scaled and translated mother wavelets. There are some example of mother wavelets such as Morlet wavelet, Paul wavelet, DOG wavelet, etc. In our study, we used the Morlet wavelet which is one of the most widely used wavelet bases. Morlet wavelet is not orthogonal but symmetric, and could be applied for continuous wavelet transform. Here is a practical guide to wavelets analysis^18^.

Wavelet analysis was used to decompose the time series of five influenza proxies into time-frequency space to reveal the seasonal patterns. However, we can only observe the periodicity and seasonality of a time series by visual inspection through wavelet analysis. To obtain the accurate seasonality parameters of peak amplitude and time, we conducted a multiple linear regression analysis with two pairs of harmonic terms based on Fourier series expansion to estimate the seasonal characteristics of influenza in Hefei, referring to the cyclic regression model in Yu’s study^8^. We fitted the model as followed:

1 $$\begin{array}{ccc}fl{u}_{i}(t) & = & ai+{b}_{i}\cdot t+{c}_{1i}\cdot cos(2\pi t/52.17)\\  &  & +\,{s}_{1i}\cdot sin(2\pi t/52.17)+{c}_{2i}\cdot cos(4\pi t/52.17)\\  &  & +\,{s}_{2i}\cdot sin(4\pi t/52.17)+{\varepsilon }_{i}(t)\end{array}$$

where ***flu***_***i***_ ***(t)*** refers to the weekly counts of the five influenza proxies as (**i** means 1 to 5) as defined, while **t** is a running index of weeks. ***a***_***i***_, ***b***_***i***_, ***c***_***1i***_, ***s***_***1i***_, ***c***_***2i***_, ***s***_***2i***_ are the coefficients, and the ***ε***_***i***_ ***(t)*** are the normal distributed errors. The linear term was used to eliminate the linear-trend influence in the time series. The harmonic terms with **T** = 52.17 and **T** = 52.17/2 respectively represent annual and semi-annual periodicities. We used 52.17 as the average weeks of a year because 2012 is a leap year during 2010 and 2015. There are 52 weeks plus one day in common years and plus two days in leap year. We calculate the average weeks as (366 + 365 ${\rm{\times }}$ 5)/6/7 = 52.1667. To get the peak time and amplitude for annual and semi-annual periodicity, we defined two indicators for each, phase.ann and phase.sem **(phase.ann = arctan(c**_**1i**_ **/s**_**1i**_ **)** and **phase.sem = arctan(c**_**2i**_ **/s**_**2i**_ **)**), amp.ann and amp.sem (**amp.ann = sqrt(c**_**1i**_^**2**^ **+ s**_**1i**_^**2**^ **)** and **amp.sem = sqrt(c**_**2i**_^**2**^ **+ s**_**2i**_^**2**^ **)**), and calculated the standard error for every indicator. Phase was used to calculate peak time, dividing phase by 2π/52.17 for annual cycle and 4π/52.17 for semi-annual cycle. To get the relative importance of semi-annual to annual cycle, we defined ratio as the proportion of the amplitude for semi-annual periodicity among the sum of annual amplitude and semi-annual amplitude (**ratio = amp.sem/(amp.ann + amp.sem)**). Confidence intervals of these indicators were calculated by block-bootstrap. We resampled 1000 datasets from the original data with replacement, each resampling with a 6-week-block bootstrap based on the weak autocorrelation between the influenza data before and after 6 weeks, which was consistent with previous research in China^14^.

## Result

### Descriptive statistics (flu%, annflu%, ILI × flu%, ILI rate, P&I)

**Figure 1:**
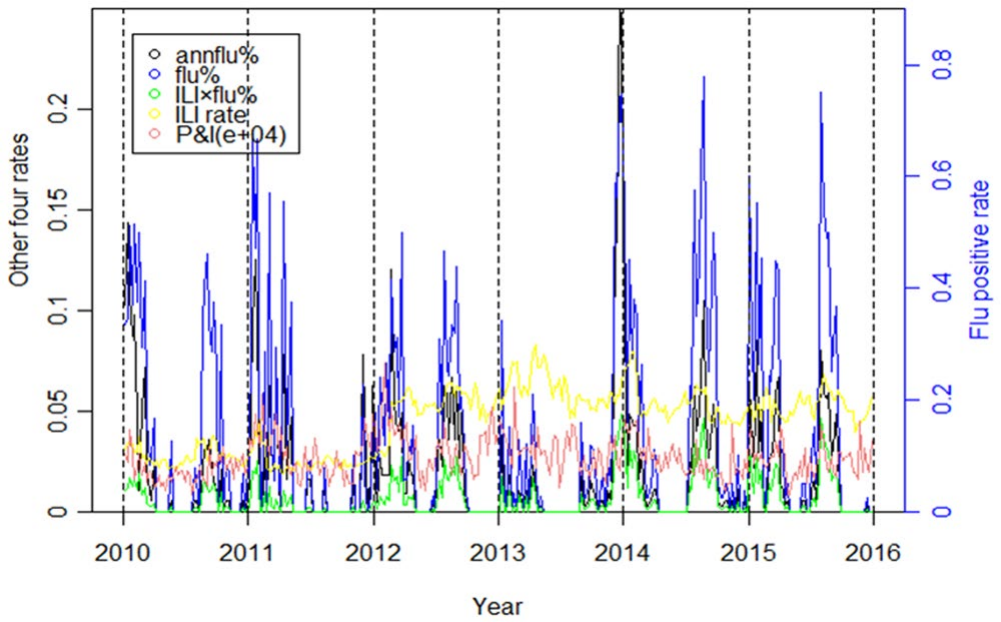
Influenza activity in Hefei, China, 2010–2015. Flu% refers to the proportion of flu positive cases among the number of cases tested per week. Annflu% refers to the proportion of flu positive cases among the sum of total flu positive cases collected per year. ILI rate refers to the proportion of ILI cases among the total outpatient patients. ILI × flu% refers to the flu% corrected with the product of flu% and ILI rate. P&I refers to the proportion of dead cases caused by pneumonia and influenza among the total citizen population.

**Figure 2:**
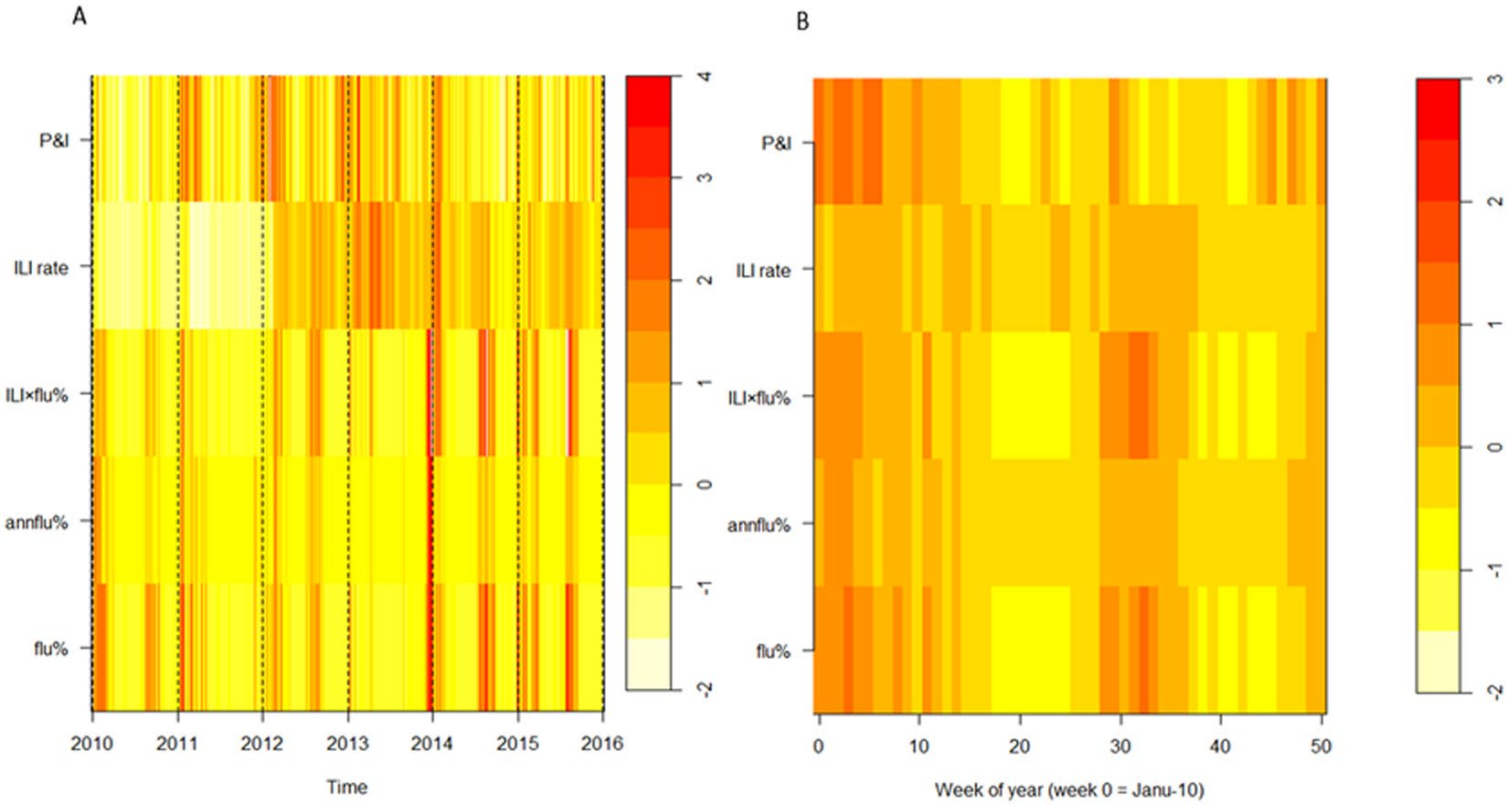
Heatmaps of influenza activity in Hefei, 2010–2015. (**A**)Time series of weekly standardized influenza cases, classed by five proxies(flu%, annflu%, ILI rate, IL × flu%, P&I) from bottom to top. (**B**) Average seasonal distribution of influenza cases, plotted as the proportion of viruses isolated in each week of a year.

Figure 1 showed that five proxies were basically synchronous in Hefei for the period 2010–2015, with peak times occurring at around January and/or July (Fig. 1). The heat-map showed that during 2010 and 2015, three flu positive rate indicators (flu%, annflu%, ILI × flu%) presented almost consistent periodicity and seasonality in semi-annual periodicity (Fig. 2). Time series of ILI consultation rate was relatively unstable and seemed increased substantially after 2012, which may result from improvement in influenza surveillance network. The P&I death rate showed annual cycle, peaking in January (Fig. 2).

### Wavelet analysis

**Figure 3:**
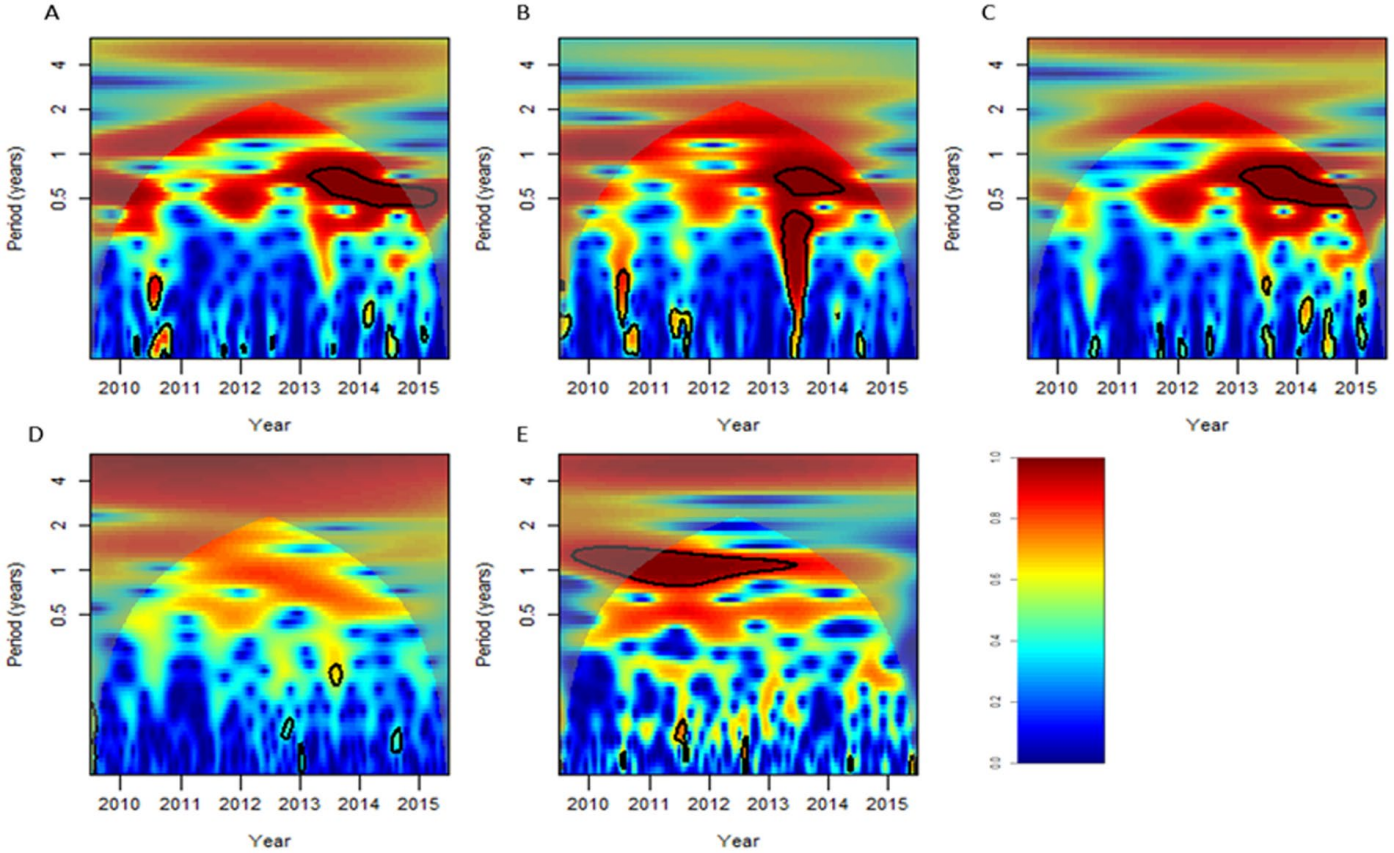
Wavelet power spectrum. The black solid line shows the regions of power significant at the 5% level computed based on 1000 Mon Carlo simulations. The corn shadow (gray area) indicated the region with edge effects. The power value of the test of seasonality were coded from dark blue for low power to dark red for high power. (**A**) flu% (**B**) annflu% (**C**) ILI × flu% (**D**) ILI rate (**E**) P&I.

The wavelets plots showed that virus positive rate indicators (flu%, annflu% and ILI × flu%) depicted semi-annual periodicity more significant than the other proxies (Fig. 3). The virus positive rate indicators (flu%, annflu% and ILI × flu%) showed a statistically significant semi-annual periodicity pattern (P < 0.05) at the years around 2013 to 2015, while not statistically significant for other years (P ≥ 0.05). While the ILI rate didn’t perform distinct periodicity during all the period. For the specific mortality indicator (P&I), it had apparent annual cycle with statistical significance.

### Seasonality regression analysis

Seasonality regression analysis provided more quantitative outcomes of seasonality characteristics, such as phase used to calculate peak time and amplitude used to calculate proportion of two kinds of periodicities. The regression fitting plot was showed in Figure S1. Table 1 showed the average peak time of the five proxies (flu%, annflu%, ILI × flu%, ILI rate and P&I) respectively at week 10.409, 12.656, 5.424, −1.236 and 12.380. The negative value of peak time means the peak of the cycle occurred at last year. The absolute value of peak time depicted by ILI rate refers to the time period from the ILI rate peak at last year to the beginning of this year. As for relative amplitude, the semi-annual cycle occupied about 40% proportion among annual and semi-annual cycles, revealed that the annual cycle has slight advantage over the semi-annual cycle in Hefei during 2010 to 2015 (Table 1).

**Table 1:** Influenza seasonal characteristics in Hefei, China, 2010–2015.

Index	Phase	Err.phase	Amplitude(95%CI)	Err.amplitude	Peek time	Odds^f^
	(95%CI)	(95%CI, E-03)		(95%CI, E-6)	(week)	
*Annual*						
flu%^a^	1.254 (−1.200,1.930)	6.642 (4.475,12.055)	0.0427 (0.008,0.089)	154.81 (46.493,239.070)	10.409	40.01%
annflu%^b^	1.524 (0.798,1.930)	1.100 (0.669,1.659)	0.0110 (0.002,0.023)	5.896 (1.853,11.473)	12.656	40.94%
ILI rate^c^	−0.149 (−0.694,2.011)	0.875 (0.705,1.069)	0.0066 (0.005,0.008)	0.215 (0.100,0.463)	−1.236	34.86%
ILI × flu%^d^	0.653 (−0.963,1.404)	0.492 (0.263,0.742)	0.0021 (0.000,0.005)	0.296 (0.067,0.579)	5.424	43.30%
P&I^e^	1.491 (1.280,1.704)	0.315 (0.264,0.383)	0.0051 (0.003,0.007)	0.524 (0.398,0.711)	12.380	43.58%
*Semi-annual*						
flu%	0.494 (−2.936,2.847)	13.035 (10.356,16.242)	0.0285 (0.0054,0.0604)	172.250 (109.78,252.68)	2.051	
annflu%	1.056 (−0.948,2.290)	2.440 (1.530,3.604)	0.0076 (0.0016,0.0169)	6.177 (2.380,12.007)	4.385	
ILI rate	0.063 (−2.270,2.360)	0.910 (0.763,1.059)	0.0035 (0.0007,0.0075)	0.840 (0.584,1.116)	0.263	
ILI × flu%	0.871 (−2.913,2.952)	0.682 (0.505,0.883)	0.0016 (0.0003,0.0038)	0.472 (0.256,0.739)	3.615	
P&I	0.657 (0.088,1.333)	0.725 (0.643,0.822)	0.0040 (0.0022,0.0058)	0.533 (0.411,0.690)	2.727	

^a^Flu% refers to the proportion of flu positive cases among the number of cases tested per week.

^b^Annflu% refers to the proportion of flu positive cases among the sum of total flu positive cases collected per year.

^c^ILI rate refers to the proportion of ILI cases among the total outpatient patients.

^d^ILI × flu% refers to the flu% corrected with the product of flu% and ILI rate.

^e^P&I refers to the proportion of the death caused by pneumonia and influenza among the total citizen population.

^f^Odds refers to the proportion of the amplitude of semi-annual cycle among the sum of the amplitude of both annual and semi-annual cycle.

**Figure 4:**
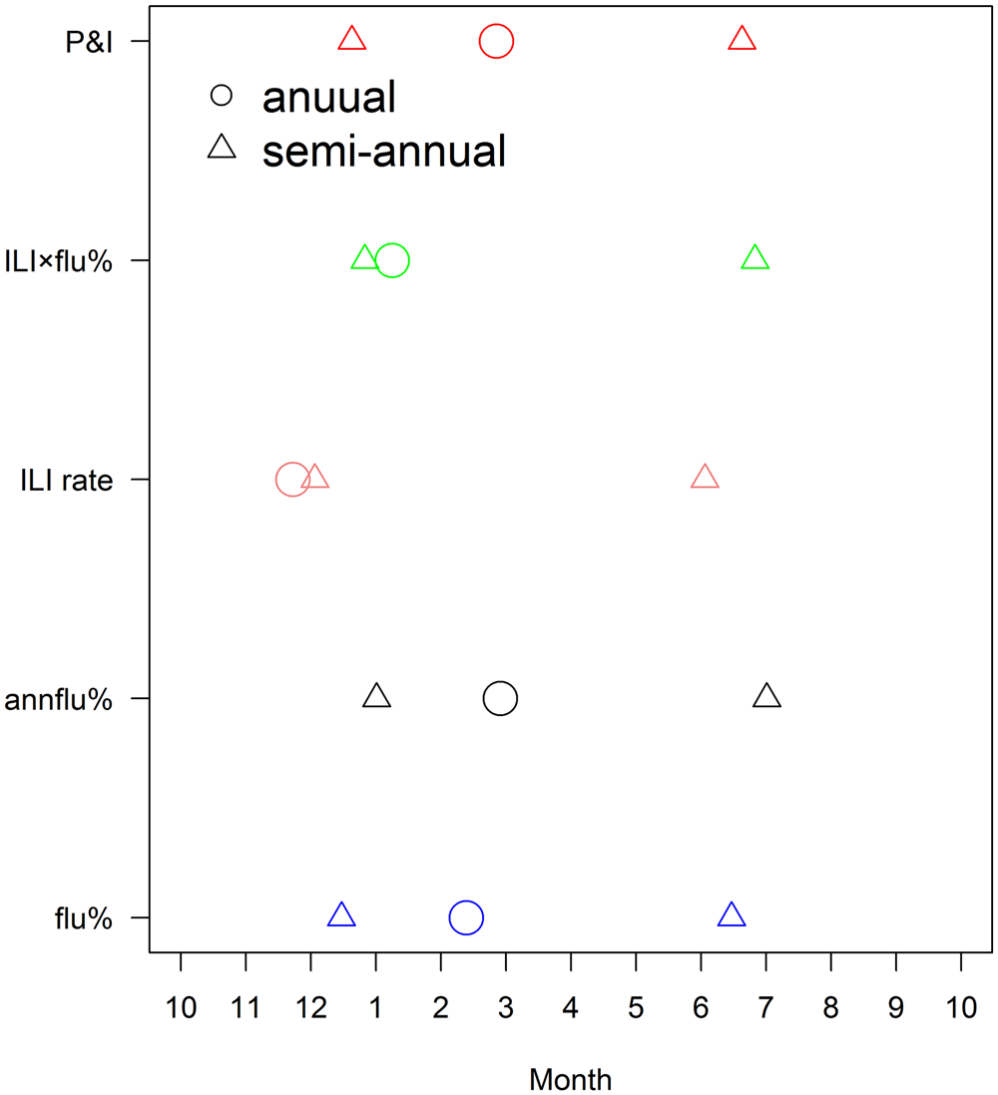
The annual peak time and semi-annual peak time for five proxies occurred in a year.

We found that the peak time usually occurred in winter (ranged from January to March) and summer (ranged from June to August) (Fig. 4). The proxy of ILI rate usually indicated influenza epidemic earlier than other proxies, while annflu% indicated influenza epidemic later than other influenza proxies (Fig. 4, Table 1). ILI rate may perform better than other influenza proxies in developing an early warning system for influenza epidemics.

## Discussion

In this study, we attempted to depict the seasonality and periodicity characteristics of influenza in Hefei with proper proxies. The heat-map and wavelet analysis indicated proxies incorporating laboratory surveillance data performed better to study influenza seasonality than other proxies based on clinical surveillance only or death registry only. The inferiority of clinical surveillance could be explained by the non-specific symptoms of influenza. Case-patients manifesting clinical symptoms of fever, cough or sore throat, may not be actually infected by influenza^19^. These symptoms could also be caused by other respiratory pathogens such as adenovirus, respiratory syncytial virus (RSV), and parainfluenza virus^11^. It revealed that flu activities proxies from laboratory testing are still more precise, which cannot be replaced by influenza-like illness proxies completely.

Among the five indicators, the pneumonia and influenza mortality performs not very well in indicating seasonality and periodicity, with only one peak in winter. Many other studies also suggest that there are caveats when using of pneumonia and influenza mortality rate as proxy for studying the peak time and amplitude of influenza activities as influenza infection is unlikely to be the sole reason for death while exacerbation of chronic conditions by influenza infection is more likely to be recorded in the death certificate^20,21^. Nevertheless, P&I may still be useful for temperate countries where influenza only peaks in winter^22,23^.

We found that Hefei has both annual and semi-annual cycles, and the proportion of annual to semi-annual cycle is likely to be 0.4. The reasons for the occurrence of two influenza epidemic peaks in some years while only has one peak in other years are unknown, and worth further investigation. In consistent with previous work on influenza seasonality in China, we found complex influenza seasonal pattern in subtropical regions. Future seasonality study at city-level in whole China will generate a more complete picture for influenza dynamic across different climate conditions.

Finally, we found that clinical surveillance of influenza indicated influenza epidemic earlier than laboratory surveillance, which is useful for developing early warning system for influenza epidemic.

Since China is a large country covering a variety of climate types. Influenza surveillance data collected in China has cultivated two seasonal cycles of influenza. Influenza in northern China possesses a regular winter peak, which is also explicit in other countries with temperate climates^1^. Some provinces in southern China are proven to cultivate semi-annual with both winter/spring and summer peaks of influenza activities^24^. The diverse patterns of seasonality make it a challenge to capture the optimal time of influenza vaccinations. The optimal time should be sufficiently early that there would be sufficient time for antibodies to rise in response to the vaccination, but not so early that protection by the vaccine wanes prior to infectious challenge^24^. Although there has always been recommendation for influenza immunity time point based on assessment of circulating virus, scientific evidence suggest that the basis for a vaccine recommendation includes not only the antigenic and genetic characteristics of the viruses but also their prevalence pattern, such as seasonality, periodicity, peak time, rate of spread, etc^25^. There haven’t been any relevant researches about influenza in Hefei during such a long period from 2011 to 2015, our research provide crucial proposal for influenza surveillance as well as the immunity program.

The combination of wavelet analysis and de-linear-trend regression which has been used separately^14,26,27^, is an innovative point of the study. On account of the findings from the descriptive statistics and wavelets analysis that the influenza activity take regular form of obvious annual and semi-annual periodicity during 2010–2015, we got more quantitative estimations from the multiple linear regression model based on Fourier series. Compared with the Serfling model and other time series model used to analyze the seasonality and periodicity of influenza activity, more visualization results are presented in our study. Additionally, we made some progresses in the definition of periodicity parameter and elimination of the linear trend in the cycle regression model according to the actual data in Hefei.

However, there are also several limitations in our research. Firstly, we do not have influenza data by type/subtype. As influenza A and B may have different seasonal pattern, future seasonality study by influenza type/subtype will provide more valuable insights regarding influenza seasonality. Secondly, no gold standard for influenza seasonality was available to compare influenza proxies. However, subtropical Hong Kong had conducted a pediatric cohort study to depict influenza seasonality and showed semi-annual cycle^28^. In combination with our findings, we suggested to use the proxies incorporating laboratory surveillance data to represent influenza seasonality in Hefei. Thirdly, the practice of collecting specimens from the first one or two ILI visits could induce the sampling bias as more severe case may come to the outpatient earlier than mild cases. However, nowadays most outpatient visits in large hospitals in China, including the two sentinel hospitals in Hefei, required appointment in advance, the sampling bias may be small. In conclusion, our study explored the seasonality and periodicity of influenza in Hefei during 2011 to 2015 using data sources from clinical surveillance, laboratory surveillance and death registry. We found that compared to proxies developed by ILI and death registration data separately, influenza proxies incorporated laboratory surveillance data performed better seasonality and periodicity, especially in semi-annual periodicity in Hefei, while influenza-like illness rate index may be more useful for development of early warning system for influenza epidemics. Annual cycle peaking in winter-spring almost occurred every year while semi-annual peak in summer only occurred in some years. Our study shows influenza seasonal pattern in subtropical China, and provides a piece of evidence for policy-makers in choosing optimal time for influenza vaccination.

## Electronic supplementary material

Supplementary information
