# Supplementary material for: Seasonal pattern of influenza activity in a subtropical city, China, 2010–2015
Source: Sci Rep. 2017 Dec 13;7:17534. doi: 10.1038/s41598-017-17806-z (PMC5727502; doi:10.1038/s41598-017-17806-z)
Supplement: Supplementary file 1 — Supplementary information [file 41598_2017_17806_MOESM1_ESM.doc]

**Supplementary information**

**Title of manuscript: Seasonal pattern of influenza activity in a subtropical city, China, 2010–2015**

**Author list:**

Xu-Xiang Liu^a,☯^, Yahong Li^b,☯^, Yibing Zhu^a^, Juanjuan Zhang^b^, Xiaoru Li^a^, Junqing Zhang^a^, Kefu Zhao^a^, Mingxia Hu^a^, Guoyou Qin^b,*^, Xi-Ling Wang^b,c,*^

^a^Hefei Center for Disease Control and Prevention, Anhui, China

^b^Department of Biostatistics, School of Public Health and Key Laboratory of Public Health Safety, Fudan University, 200231 Xuhui District, Shanghai, China

^c^Shanghai Key Laboratory of Meteorology and Health, Shanghai, China

☯Authors contribute equally to this work.

*Correspondence to: Dr Xi-Ling Wang, Email: [erinwang@fudan.edu.cn](mailto:erinwang@fudan.edu.cn), Tel: 86-21-33562913 or Dr Guoyou Qin, Email: [gyqin@fudan.edu.cn](mailto:gyqin@fudan.edu.cn), Tel: 86-21-33563917


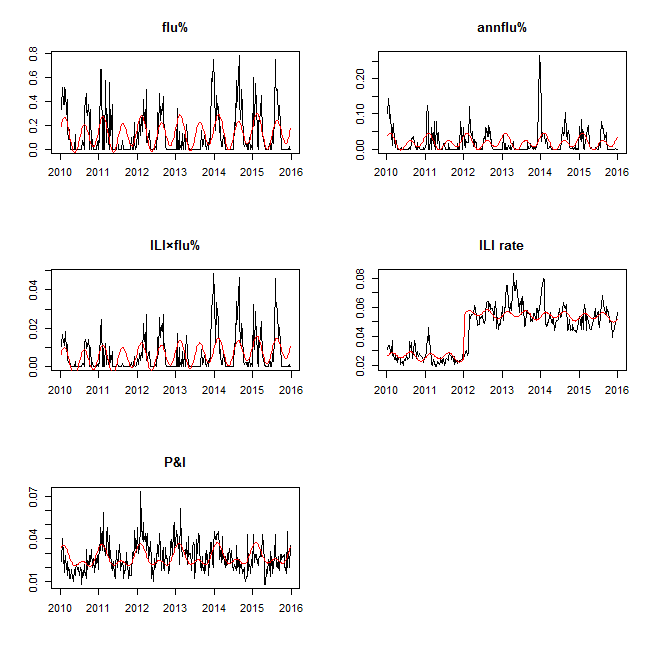


**Supplementary Figure S1 The regression fitting plots of five proxies.**
